# Pathophysiological, immunological, and inflammatory features of long COVID

**DOI:** 10.3389/fimmu.2024.1341600

**Published:** 2024-02-28

**Authors:** Karen Bohmwald, Benjamín Diethelm-Varela, Linmar Rodríguez-Guilarte, Thomas Rivera, Claudia A. Riedel, Pablo A. González, Alexis M. Kalergis

**Affiliations:** ^1^ Millennium Institute on Immunology and Immunotherapy. Facultad de Ciencias Biológicas, Pontificia Universidad Católica de Chile, Santiago, Chile; ^2^ Instituto de Ciencias Biomédicas, Facultad de Ciencias de la Salud, Universidad Autónoma de Chile, Santiago, Chile; ^3^ Millennium Institute on Immunology and Immunotherapy, Departamento de Ciencias Biológicas, Facultad de Ciencias de la Vida, Universidad Andrés Bello, Santiago, Chile; ^4^ Departamento de Endocrinología, Facultad de Medicina, Pontificia Universidad Católica de Chile, Santiago, Chile

**Keywords:** SARS-CoV-2, long Covid, epidemiology, pathophysiology, neurological alterations, immune response.

## Abstract

The COVID-19 pandemic continues to cause severe global disruption, resulting in significant excess mortality, overwhelming healthcare systems, and imposing substantial social and economic burdens on nations. While most of the attention and therapeutic efforts have concentrated on the acute phase of the disease, a notable proportion of survivors experience persistent symptoms post-infection clearance. This diverse set of symptoms, loosely categorized as long COVID, presents a potential additional public health crisis. It is estimated that 1 in 5 COVID-19 survivors exhibit clinical manifestations consistent with long COVID. Despite this prevalence, the mechanisms and pathophysiology of long COVID remain poorly understood. Alarmingly, evidence suggests that a significant proportion of cases within this clinical condition develop debilitating or disabling symptoms. Hence, urgent priority should be given to further studies on this condition to equip global public health systems for its management. This review provides an overview of available information on this emerging clinical condition, focusing on the affected individuals’ epidemiology, pathophysiological mechanisms, and immunological and inflammatory profiles.

## 1 Introduction

The ongoing coronavirus disease 2019 (COVID-19) pandemic, caused by the severe acute respiratory syndrome coronavirus 2 (SARS-CoV-2) (1), has resulted in over 771 million confirmed cases and more than 6.9 million deaths globally (2). It has also led to significant morbidity, adverse mental health outcomes (3–7), and substantial socioeconomic disruption (8–11). While efforts have primarily focused on the acute phase of the disease, emerging evidence of significant and concerning post-acute effects on COVID-19 survivors introduces an additional dimension to the pandemic, potentially with long-term implications. These post-acute effects are known as long COVID or by various other names such as post-COVID conditions (PCC), post-acute sequelae of COVID-19 (PASC), chronic COVID syndrome (CCS), and long-haul COVID, and they define a broad spectrum of lingering symptoms experienced by a proportion of COVID-19 survivors after clearing the acute SARS-CoV-2 infection (12). According to the United States (US) Centers for Disease Control and Prevention (CDC), post-COVID-19 conditions have various health consequences four or more weeks after SARS-CoV-2 infection. The term is loosely defined due to the diverse and heterogeneous nature of the long COVID symptoms. These may include respiratory issues like persistent breathlessness, dyspnea, tiredness, headache, fever, and multi-system complaints. Additionally, these symptoms may occur concurrently or intermittently (12, 13). Nevertheless, in certain instances, the clinical manifestations observed almost four weeks following SARS-CoV-2 infection might derive from persistent symptoms originating in the acute phase of COVID-19 (14). In light of this, the significance of defining long COVID symptoms to distinguish between patients experiencing a gradual recovery and those exhibiting symptoms that accurately align with their medical condition, has been proposed (14).

This review provides a comprehensive overview of long COVID’s clinical and biological aspects. It emphasizes the epidemiology, clinical presentation, and system-specific manifestations, focusing on the immune response and inflammation.

## 2 Epidemiology of long COVID

Following the 2003 SARS epidemic caused by SARS-CoV-1, some survivors experienced reduced functional capacity and could not return to work (15, 16). The prevalence of long COVID in the population remains uncertain, ranging from 10% to 60% for COVID-19 survivors and potentially exceeding 90% for severe cases requiring hospitalization (17–23). It is estimated that one-fifth to one-third of survivors may experience activity-limiting symptoms for weeks after the acute phase (20, 21, 24–28). Notably, post-COVID-19 persistent symptoms appear more common than other respiratory infections (19, 29). Studies in Italy found that most patients surviving COVID-19-related hospitalizations faced impaired physical functioning after discharge (19).

**Figure 1:**
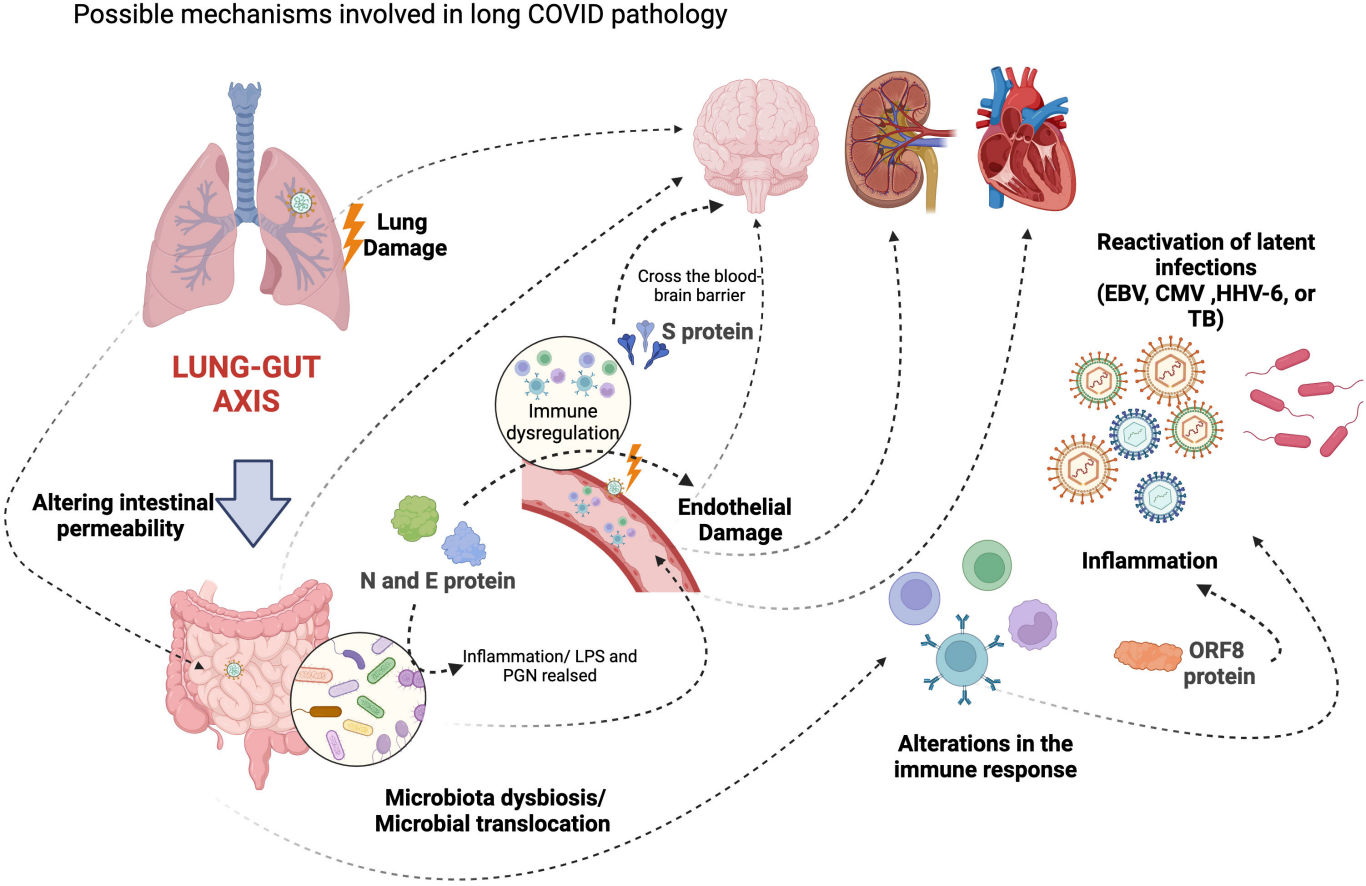
Possible mechanisms involved in the pathologies associated with long COVID. The figure summarizes the possible mechanisms involved in long COVID pathology. Immune dysregulation, autoimmunity, endothelial dysfunction, and hidden viral persistence in various organs are possible underlying pathophysiological mechanisms involved in long COVID. Viral persistence can lead to intestinal microbiota dysbiosis, which alters the immune response. It has been reported that changes in the intestinal microbiota can lead to neurological alterations. On the other hand, the spike protein could cross the blood-brain barrier, contributing to neurological sequelae. Additionally, nucleocapsid and envelope proteins can cause damage to endothelial cells, which can induce cytokines and activate immune responses. In addition, immune dysregulation can reactivate other viruses, such as EBV, HHV-6, and CMV, and bacterial diseases, such as TB. (Created with BioRender; License # IY26FE9X7C).

Furthermore, a case series of 143 COVID-19 survivors found that only 12.6% were symptom-free after an average of 60 days from disease onset (27). Additionally, 44.1% reported a declining quality of life due to fatigue and dyspnea (27) (**Figure 1**). Long-term COVID development can be influenced by different SARS-CoV-2 variants, the number of vaccine doses received, and the time elapsed since vaccination (30). Studies indicate that Omicron cases were less likely to experience long COVID (31, 32). For instance, a study in China reported that 40.4% of patients with the Delta variants had clinical sequelae 3 to 24 months after discharge, compared to only 8.89% who had Omicron (32). Similarly, a study in the United Kingdom showed a reduction in long COVID cases from 10.8% for the Delta variant to 4.5% for the Omicron variant (31). Moreover, Omicron cases were less likely to experience long COVID regardless of their vaccination regimen (31, 32). However, early studies on this matter often had limited sample sizes and focused on specific populations, so further research is needed to reach more definitive conclusions.

The health agencies estimate that around 1 out of 5 COVID-19 survivors may develop long COVID, marking some of the most robust evidence of its prevalence thus far (22). The CDC conducted a multi-state telephone survey of symptomatic adult COVID-19 survivors in the US to assess the prevalence of disabling or activity-limiting symptoms after COVID-19. The survey found that 35% had not returned to their normal state of health after 2–3 weeks of diagnosis. It has also been reported that one in five patients aged 18–34 with no chronic medical conditions after COVID-19 did not return to their normal state of health (29). While information on the duration of long COVID is limited, reported cases have extended from weeks to over a year (18, 33). However, recent studies indicate a decline in the incidence of long COVID cases post-pandemic, and this reduction has been associated with the advancement of vaccination (34, 35) Additionally, the Wuhan variant has been associated with diverse physical symptoms and emotional and behavioral changes, indicating a substantial impact on long-term health outcomes (36). In contrast, the Omicron variant has shown fewer post-infection effects, resembling common seasonal viral illnesses. These observations suggest that the Omicron and subsequent variants may not have the same long-term health consequences as earlier SARS-CoV-2 variants (36).

Certain risk factors, including age, gender, cardiovascular disease, and diabetes, have been associated with long COVID (37–43), which are also risk factors for severe acute COVID-19 (44, 45) Although some studies suggested that long COVID incidence may correlate with COVID-19 disease severity, age, and comorbidities (38, 46–48), others did not show such a relationship (23, 49). Notably, long COVID can emerge after mild and even asymptomatic COVID-19 cases (50). Observational evidence indicates that the severity of long COVID symptoms does not necessarily correlate with COVID-19 disease severity (50). This insight is derived from a study utilizing data from the UK Biobank, an extensive health database with measurements from thousands of volunteers before and after the pandemic’s start (51). On the other hand, an association between disease severity and the intensity of lingering respiratory symptoms has also been reported (52). Available information suggests a possible link between COVID-19 severity and long COVID incidence (53), although this association may not be statistically significant in large-scale studies and thus requires further investigation.

Female gender has been identified as a risk factor for developing post-COVID-19 symptoms (54–58). Studies indicate that women (<50 years old) were more likely to report fatigue and dyspnea after acute infection compared to men of the same age (55, 56, 59). Additionally, women may be up to three times more likely to be diagnosed with long COVID, regardless of age (60). This could be attributed to biological differences in the expression of angiotensin-converting enzyme-2 (ACE2) and transmembrane serine protease 2 (TMPRSS2) between genders (61), as well as immunological variations (62).

The role of persistent infection in long COVID remains uncertain. Various hypotheses regarding the mechanisms involved in its pathogenesis have been proposed, including circulating viral antigens or reservoirs in tissues (**Figure 1**) (30). Clinical and epidemiological surveillance data show that individuals may have detectable viral loads yielding positive PCR results for three months or even longer after infection. Although these individuals are usually non-infectious, their persistently detectable SARS-CoV-2 genetic material may contribute to their long-term symptoms (63–65). On the other hand, emerging research has reported the detection of persistent circulating spike protein in the bloodstream of some long COVID patients, even though they have undetectable levels of viral genetic material after the acute phase of COVID-19 (66). It has been suggested that the spike protein may go across the blood-brain barrier, triggering inflammation (67). Moreover, the persistence of nucleocapsid and envelope proteins in the liver, gallbladder, lymph nodes, and intestine has been associated with endothelial cell damage, leading to cytokine release and immune response activation (68).

Although chronically SARS-CoV-2-positive patients are generally considered non-infectious, persistent viral shedding has been reported (24). This poses a concerning risk of potential new variant emergence (69, 70). Chronic infections have led to mutations that could facilitate SARS-CoV-2 evolution and the emergence of new variants (70–73). As a result, epidemiologists and infectious disease specialists have hypothesized that SARS-CoV-2 variants like Omicron may have originated through natural selection in chronically infected hosts (69). Emerging observational data suggests that vaccination against COVID-19 may help resolve chronic infections, emphasizing the potential of vaccines in preventing the emergence of new variants (72, 74). It is important to note that while the quality of the evidence is somewhat limited, it indicates a positive impact of vaccinations in mitigating the ongoing emergence of variants.

Since the widespread availability of COVID-19 vaccines in early 2021, extensive scientific scrutiny of their impact on the risk of developing long COVID and the immune profile following a post-breakthrough SARS-CoV-2 infection (15, 54, 75–89). Studies have also investigated the severity of symptoms in vaccinated individuals who develop long COVID (82–86). Initial reports have suggested that vaccination may confer protective effects against long COVID incidences (83, 85, 90). However, recent extensive cohort studies show that vaccines provide only partial protection in case of a breakthrough infection (91, 92). One of these studies, conducted using the US Department of Veteran Affairs healthcare database with substantial sample size, provides robust evidence that vaccination’s impact in reducing the incidence of long COVID is partial (82). Available data conclusively demonstrate that vaccines diminish the risk of long COVID by decreasing the likelihood of experiencing a SARS-CoV-2 infection (84, 87, 90). Other studies have shown an almost complete reduction of long COVID symptoms after vaccination, mainly for severe manifestations like fatigue (93, 94). In this context, booster doses may be considered a preventive measure against long COVID, helping to maintain a relatively low infection risk and providing protection against concerning variants (86, 95, 96).

The FDA-approved antivirals Nirmatrelvir and Molnupiravir effectively treat acute COVID-19 (97, 98). Nirmaterlvir targets the SARS-CoV-2 type 3-chymotrypsin cysteine protease enzyme (99), while Molnupiravir heightens the frequency of viral RNA mutations (98), both leading to alterations in viral replication (41, 98). Administering these antivirals during the acute phase is linked to a reduced risk of at least eight of the thirteen post-acute sequelae, including arrhythmia, pulmonary embolism, deep vein thrombosis, fatigue and malaise, liver disease, acute kidney injury, muscle pain, and neurocognitive impairment (100, 101). Their use is associated with decreased risk of long COVID in unvaccinated individuals, vaccinated individuals, and those with both a primary SARS-CoV-2 infection and reinfection (100, 101).

Immunocompromised individuals show a notably diminished humoral immune response to vaccination, underscoring the necessity of booster shots in averting COVID-19 and mitigating the onset of long COVID within this group of patients (79). In immunocompromised children, SARS-CoV-2 infection was observed to be more asymptomatic than their immunocompetent counterparts (102). Moreover, there was a lower reported frequency of long COVID among immunocompromised children relative to immunocompetent controls (102). This is crucial, especially given the limited but strong evidence suggesting that persistent infection in these individuals may contribute to the emergence of new variants, as discussed earlier (69–72). However, it is worth noting that additional protection in the case of a breakthrough infection remains a subject of scientific debate. Positively, evidence indicates that vaccination may alleviate long COVID symptoms in previously unvaccinated patients (74, 83, 93, 94, 103, 104).

Long COVID is not exclusive to adults, as it has also been observed in the pediatric population (105). For example, a retrospective cohort study found that 51% of pediatric patients from a sample of children who recovered from COVID-19 experienced persistent symptoms 1-3 months after infection. The most common manifestations in this study were fatigue, loss of taste or smell, and headaches (106). Similarly, a small case report involving Swedish children who experienced mild to moderate COVID-19 without hospitalization revealed that all five children had persistent symptoms after 6-8 months, significantly hindering their daily activities and severely impacting their ability to attend school (107).

## 3 Pathophysiology of long COVID

**Figure 2:**
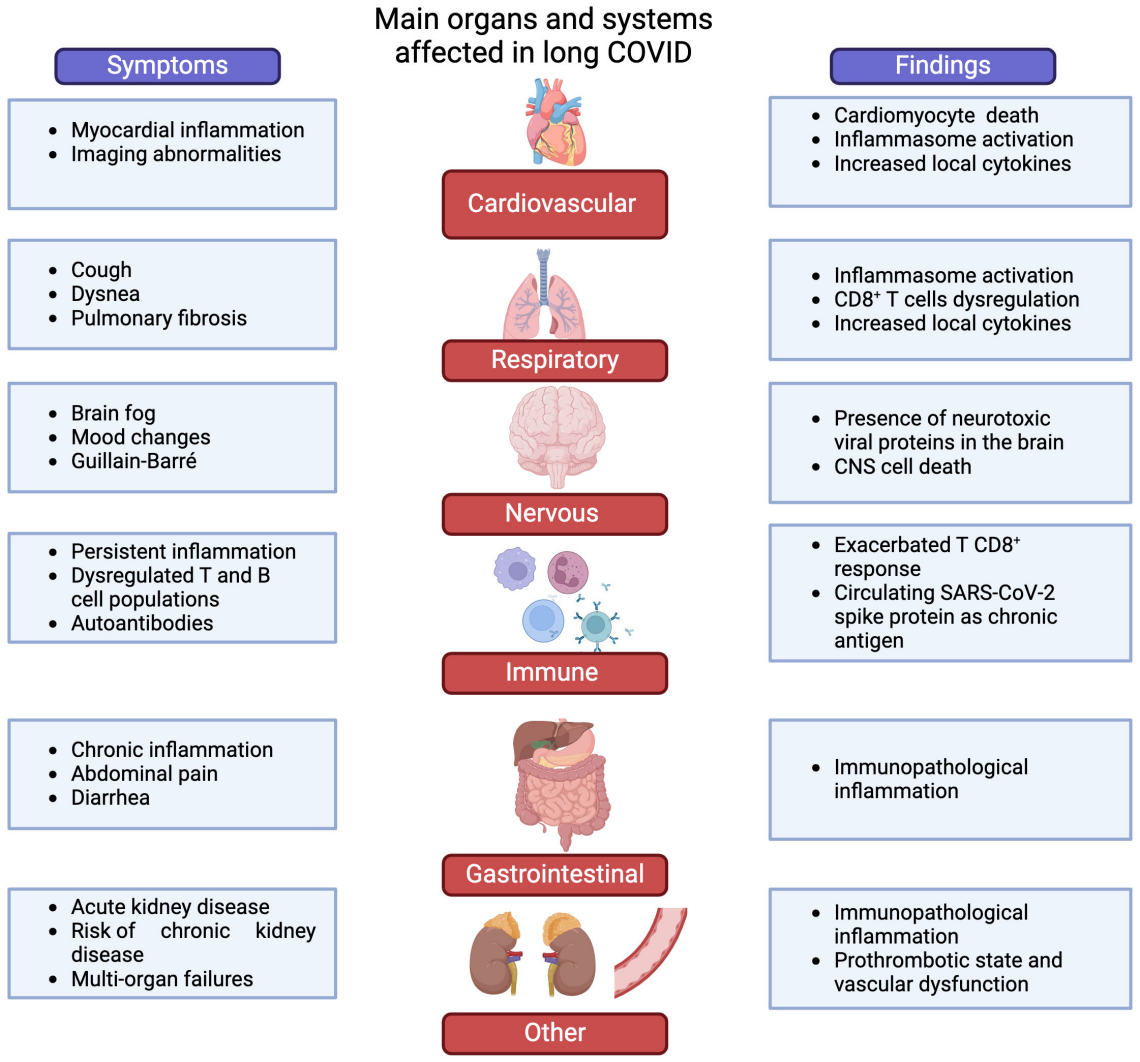
Main systems involved in long COVID. The figure summarizes common complications and some putative mechanisms involved in long COVID. It is estimated that 1 in 5 COVID-19 survivors develop long-lasting symptoms compatible with long COVID, which are diverse and heterogeneous. These complications can be classified into six main groups: cardiovascular, respiratory manifestations, neurological, alterations in immunity, gastrointestinal manifestations, and others. The mechanisms underlying these complications related to long COVID are still poorly understood, and relevant findings are summarized on the right side of the figure. (Created by BioRender; License # QU26HKW75S).

Long COVID presents a wide range of symptoms, as mentioned before (**Figure 2**) (12, 13, 22, 108, 109). Despite its clinical presentation, the precise pathophysiological mechanisms remain unclear. Its involvement across multiple organs suggests potential mechanisms, including direct viral tropism, cytotoxic viral proteins, and immunopathology (**Figure 2**). Some studies propose that long COVID symptoms may result from persistent effects of the acute phase of COVID-19 and that different mechanisms may cause the different symptoms described (14, 27, 66, 67). Additionally, immune system activation can lead to an autoimmune response or prolonged, nonspecific inflammation, causing further harm to host cells (**Figure 2**) (24, 110–115).

### 3.1 Clinical presentation of the disease

The CDC lists common long COVID symptoms, including dyspnea, fatigue, post-exertional malaise, cognitive impairment, mood changes, cough, chest pain, headache, fever, lightheadedness, anosmia, and dysgeusia (13). Like the acute phase, long COVID exhibits a multi-systemic nature (108, 109, 116). Two primary symptom patterns typically emerge in long COVID cases (1): fatigue and upper respiratory symptoms and (2) multi-system symptoms (28, 43). Due to the diverse presentation of this condition, establishing precise, standardized symptoms remains challenging. However, insights from patient follow-up studies have been invaluable. An investigation revealed enduring manifestations, primarily including breathing difficulties, fatigue, muscle weakness, anxiety, and depression, with fatigue or muscle weakness affecting a significant majority (63%) of survivors. However, respiratory and multi-organ complications described in long COVID do not appear to be linked to the need for mechanical ventilation (MV) during the acute phase (117). Some authors even report complete patient recovery one year after the acute phase, even in those patients with worse respiratory mechanics during invasive MV (117). However, other studies have shown that 60 to 70% of neuropsychiatric symptoms persisted between 3 and 6 months in patients who received MV (118, 119). The emergence of psychiatric symptoms, including psychological distress, anxiety, depression, sleep disorders, and alterations, has been documented in survivors of severe illnesses (120), including those recuperated from severe infections with other coronaviruses (121, 122). These symptoms appear to be linked with post-traumatic stress, potentially stemming from the isolation these patients experienced (123). During SARS-COV-2 infection, patients supported with MV may also experience paresthesia, neuropathies, and mobility limitations, which could be connected to the loss of gray matter during the acute phase (48). It is important to note that factors, such as MV or the socioeconomic status of patients, independent of viral infection, can contribute to symptoms like anxiety and depression (124, 125).

Despite the challenges mentioned above, specific long COVID symptoms correlate with measurable clinical indicators, including mild multi-organ impairment, mainly affecting the heart, liver, and pancreas. This discovery raises concerns about potential chronic organ damage, emphasizing the need for preventive strategies and additional research to mitigate such harm. Implementing grading scales and quantification tools for evaluating long COVID symptoms in patients could offer a potential solution to enhance follow-up and improve outcomes.

Scientists have devised strategies to assess and categorize the effects of long COVID. One such tool is the post-COVID-19 functional status (PCFS), an ordinal system that evaluates the condition of COVID-19 survivors over time (126). It employs a 4-grade scale based on functional limitations, ranging from grade 1 (indicating no limitations) to grade 4 (indicating dependence on external care for survival). This tool has demonstrated reproducibility in identifying patients needing support and rehabilitation (126). Since its introduction, it has been utilized in clinical studies on long COVID (127) and independently validated (128). A core outcome set (COS) has been proposed as a clinical instrument to establish consistent clinical outcomes (129). A multinational effort has created a COS for long COVID using a systematic literature review and a modified Delphi process. This COS is designed for adult patients in various settings (129–131). It encompasses outcomes such as fatigue or exhaustion, pain, post-exertion symptoms, work or occupational and study changes, survival from acute disease, symptoms related to physical functioning, cardiovascular and nervous system symptoms or conditions, and mental and respiratory functioning (130). Due to its global applicability and rigorous methodology (130), this COS is strongly recommended for evaluating long COVID patients in clinical and research settings.

Moreover, several studies have employed established grading scales to assess quality of life, disability, and specific levels of impairment in various tissues and physiological systems among long COVID patients (26). These tools include the EuroQol 5-Dimension 5-level (EQ5D-5L) instrument for assessing quality-of-life (132), the Washington Disability Group (WG) Short Form (133), 6-minute walking distance (127), and several clinical scales for dyspnea and fatigue (127). It is relevant to mention that quantifying the severity of long COVID is crucial for differentiating individuals experiencing minor lingering symptoms from those significantly impaired. This categorization aids in selecting participants for studies and prioritizing medical interventions.

As the pandemic endures, we anticipate gaining more insights into patient cases, providing a clearer understanding of this condition. For example, a case series focusing on female patients with long COVID symptoms revealed a notably high prevalence of orthostatic intolerance syndrome. This prompted the authors to suggest that autonomic nervous system dysfunction, potentially driven by autoimmune inflammation or viral invasion, could be an underlying factor (134). A 2020 cross-sectional study examined patients four to six weeks post-discharge from the hospital following COVID-19. It documented persistent respiratory symptoms and fatigue in many survivors (135).Elevated levels of inflammation markers, including d-dimer and C reactive protein, were also noted (135). The sustained inflammation observed in long COVID patients may have broader implications, given SARS-CoV-2’s capacity to activate inflammatory responses and oncogenic pathways. There is speculation that individuals with long COVID may face an increased risk of developing cancer (136) .

### 3.2 Respiratory consequences associated with long COVID

SARS-CoV-2 is linked to enduring respiratory issues, such as pulmonary fibrosis, angiogenesis, and vessel remodeling (91, 137–140). Interestingly, the seriousness of respiratory problems seems connected to the neurological effects of COVID-19 (141). In long COVID, common respiratory complications comprise breathlessness, dyspnea, fatigue, and a lingering cough, most of them at four weeks after diagnosis (26, 142). Estimates for the persistence of post-COVID-19 cough vary in studies and meta-analyses, generally falling between 7% to 15% for non-hospitalized and hospitalized survivors, regardless of whether they required MV (47, 143, 144). Breathlessness has been observed more frequently in individuals hospitalized at least one year after COVID-19, and these patients present lower forced expiratory volume in 1s than long COVID patients without breathlessness (145, 146). The secondary inflammation caused by the viral infection provokes a more significant overflow obstruction in these patients. The lung injury observed in long COVID patients can be mediated by an abnormal migration of monocytes, which, during the acute phase of the infection, had increased expression of C-X-C chemokine receptor 6 (CXCR6) and remained long-term elevated (146).

On the other hand, the prevalence of fatigue can vary between 32-42% in the first six months after COVID-19. Meanwhile, dyspnea has been reported between 12-26% of the long COVID cases (147, 148). Notably, not all long COVID patients had these symptoms during the acute phase of the disease. The risk factors associated with these symptoms are the female gender and having them during the first infection (148).

Soluble markers of inflammation are elevated in long COVID patients, which include general markers like C-reactive protein, components of the complement complex, and inflammatory cytokines such as interleukin-6 (IL-6), IL-1α, transforming growth factor beta (TGF-β), chemokines, interferon (IFN)-β, IFN-γ, granulocyte colony-stimulating factor (GM-CSF), as well as autoantibodies (40, 113, 138). These findings indicate that long COVID may primarily involve inflammation. However, long COVID patients with severe lung fibrosis have been found to have lower IFN-β production compared to those without fibrosis (138). Clinical studies consistently demonstrate increased local inflammation markers aforementioned in both severe COVID-19 patients and long COVID patients with lung fibrosis (138, 149). Another study found elevated levels of several inflammatory cytokines, including TGF-β but not IFN-β, in long COVID patients with lung complications like pulmonary fibrosis (138). Similarly, an analysis of post-mortem samples from COVID-19 cases indicated elevated type-I IFN response in lung tissue due to SARS-CoV-2 infection (149). Considering its potential link to inflammation, glucocorticoid therapy could be beneficial for managing long COVID (135, 150). Furthermore, prolonged inflammation may contribute to developing long COVID, as shown by an upregulated cytokine secretome in post-acute COVID-19 survivors (151). Another study reported similar findings regarding the decline of IFN-γ-producing CD8^+^ T lymphocytes in long COVID patients (24). SARS-CoV-2 might utilize mechanisms to suppress the host’s type-I IFN response, which persists long after infection clearance, and this diminished type-I IFN may play a role in the persistently exacerbated inflammation (24, 152). Additionally, inflammasome activation is emerging as a critical driver of chronic COVID-19 symptoms, affecting respiratory physiology. This prompts further investigation into inflammasome modulation as a potential intervention (153–156).

It is worth noting that mechanical ventilation (MV) in non-COVID-19 pathologies has been reported to lead to respiratory complications similar to those seen in long COVID, such as barotrauma (damage to the body caused by air or water change*)*, volutrauma (lung injury caused by excessive stretching of the alveoli due to high tidal volumes during MV), and pneumothorax (presence of air or gas in the pleural cavity, leading to lung collapse) (157–160). Mechanical ventilation-induced lung injury can generate a systemic inflammatory response affecting other organs, also reported in long COVID (161). Moreover, MV seems to contribute to the development pulmonary fibrosis in elderly patients who have recovered from COVID-19 (162). These reports suggest that MV plays a role in the respiratory complications observed in long COVID patients.

### 3.3 Findings related to neurological alterations

COVID-19 can have lasting neurological effects in up to 30% of hospitalized patients at 3 months post discharge (25), often marked by “brain fog,” including disorientation, dizziness, and difficulty concentrating, sometimes leading to disability (**Figure 2**) (163). Termed “neuro-COVID” by some experts (164, 165) SARS-CoV-2 joins a list of viruses linked to neuropsychological issues (166), including the human respiratory syncytial virus (hRSV), human immunodeficiency virus (HIV), other human coronaviruses, Zika virus, herpes simplex virus (HSV), human cytomegalovirus (HCMV) and influenza (166–169). There is concern about potential neurological damage to offspring from infections in pregnant women, possibly leading to future congenital “neuro-COVID” manifestations (170). Neurological complications have been tied to the severity of respiratory infections (16), hospital stays (171) and exacerbated immune responses (172), potentially contributing to some “neuro-COVID” symptoms (16, 166, 167, 173, 174).

Similar to acute COVID-19, sudden loss of smell and taste (anosmia and ageusia) is prevalent in long COVID, initially experienced by around 15% of COVID-19 patients (175). While most recover these senses within weeks, these symptoms may persist in long COVID cases (176). Persistent loss of smell and taste has been linked to direct viral harm to olfactory receptor neurons and immune system-induced damage (5).

To understand cognitive impairment in long COVID, examining acute COVID-19 findings is relevant. A study of 81,337 volunteers showed a significant association between symptomatic COVID-19 and reduced cognitive function, especially in cases involving MV (141). MV alone, even without COVID-19, can cause neurological complications, which may contribute to long COVID neurological manifestations (119, 177). Even fully recovered individuals displayed poorer cognitive performance than uninfected counterparts, indicating a link between symptomatic COVID-19 and cognitive impairment (141).

An extensive study utilizing the UK Biobank repository (50) found significant correlations between COVID-19 infection and brain imaging abnormalities, even in mild cases (48). A recent cross-sectional study identified neurocognitive impairments in COVID-19 survivors months after infection, impacting functioning and quality of life and correlating with disease severity and comorbidities (178).

The exact pathophysiological mechanisms behind SARS-CoV-2-induced neurological alterations remain unclear. Nevertheless, emerging evidence suggests the virus breaches the blood-brain barrier (BBB) and directly damages the central nervous system (CNS), possibly triggering neuropsychological effects as seen in severe cases or post-mortem findings (179–182). Besides the elevated local inflammation, the direct invasion potentially accounts for the neuropsychological aftermath experienced by numerous COVID-19 survivors (**Figure 2**). This phenomenon could provoke a difunctional neurological signaling leading to the symptoms observed in long COVID cases (**Figure 1**). Notably, coronaviruses, including SARS-CoV-1, have been noted for their neurotropic tendencies, supporting the plausibility of direct neurological invasion by SARS-CoV-2 (142, 179, 181, 183). SARS-CoV-2’s presence in the CNS, extracted from cerebrospinal fluid during pandemic studies, might contribute to enduring neuropsychiatric complications (184). Animal models indicate that neurological coronavirus invasion could lead to long-lasting neurological repercussions in humans (185, 186). These models could partially elucidate the lingering cognitive impairment in many long COVID cases in humans.

An additional approach to elucidate the mechanism of SARS-CoV-2-induced neurological damage lies in identifying cytotoxic proteins expressed by the virus (5, 187). An analysis of SARS-CoV-2 proteins found some—specifically ORF6 and ORF10—possessed amyloidogenic properties and highly cytotoxic effects in neurons, potentially underpinning neurological alterations in long COVID and posing a risk for neurodegenerative disease (188).

Recently reported cases following SARS-CoV-2 infections include acute sensory and motor axonal neuropathy and acute inflammatory demyelinating polyradiculoneuropathy (**Figure 2**) (165, 189–191). These are subtypes of Guillain-Barré syndrome (GBS), where exposure to foreign peptides resembling those in peripheral nerves can lead to neuropathy (192, 193). Interestingly, SARS-CoV-2 contains hexapeptides, rich in lysine (KDKKKK) and glutamic acid (EIPKEE) amino acids, resembling human heat shock proteins 60 and 90 (192), potentially triggering an autoimmune response and neuropathy (5, 49, 141, 178, 194). It is important to mention that it is possible that neurological manifestations can be due to an immune pathology and not to CNS viral infection.

### 3.4 Cardiovascular complications found in patients with long COVID

Cardiovascular issues are recognized among the potential outcomes in long COVID cases (12, 13). Symptoms encompass chest pain, postural tachycardia syndrome, and left or right ventricular dysfunction, among other clinical signs (195). Additionally, irregularities in electrocardiograms (ECG) and Holter-ECG, temporary or lasting, have been observed in specific long COVID individuals, occurring in 1% to 27.5% of patients hospitalized due to cardiovascular problems (195). Another study revealed that the risks and associated burdens were apparent, particularly among individuals not hospitalized during the acute phase of the disease—this category comprising most COVID-19 cases. Furthermore, it was noted that the risks and associated burdens demonstrated a gradual escalation across the severity spectrum of the acute phase of COVID-19, ranging from non-hospitalized individuals to those requiring hospitalization and eventually those admitted to intensive care (196). Most cardiovascular issues noted in long COVID align with observations from the acute phase, including myocardial injury (**Figure 2**) (197, 198)), with cardiovascular disease emerging as a severe disease risk factor. Research reveals that 24.4% of hospitalized COVID-19 patients faced cardiac injury, which is strongly linked to higher mortality rates (199). These cardiovascular complications might persist in some patients post-recovery (198). Studies examining cardiac impairment after COVID-19 recovery found that 78% of survivors displayed cardiovascular magnetic resonance (CMR) abnormalities, with 60% showing persistent myocardial inflammation (200). These findings are noteworthy alongside evidence of COVID-19’s direct invasion of heart tissue (201) and *in vitro* studies replicating myocarditis (202). While mechanisms behind sustained heart inflammation remain partly unknown, overactive innate immune responses, like those in COVID-19-related lung damage, are suggested as plausible causes (156). The prevalence of these findings highlights the likelihood of COVID-19 survivors coping with cardiovascular issues, emphasizing the need for extended efforts in diagnosis and tailored management.

Histological and molecular findings from severe COVID-19 cases indicate significant local heart inflammation, suggesting a potentially similar condition in individuals with long COVID, leading to lasting myocardial injury (149, 195, 203). Heart inflammation has been linked to COVID-19 symptoms and mortality (204, 205). However, not all data confirm a significant prevalence of myocarditis in patients with sustained cardiovascular symptoms post-COVID-19 (206).

While persistent myocardial inflammation indicates long COVID, in some cases, comprehensive research is crucial to assess this population’s underlying pathophysiological mechanisms and cardiovascular risk profile. Investigations into post-COVID-19 cardiovascular damage remain crucial for a deeper understanding of adverse cardiovascular outcomes among survivors. Additionally, the vascular endothelium’s role in connecting inflammation and coagulation contributes to a prothrombotic state and vascular dysfunction in long COVID subjects (207).

### 3.5 Damage to endothelial barriers and viral persistence

The endothelium, crucial in hemostasis, immune reactions, and angiogenesis, plays a pivotal role in SARS-CoV-2 infection (208). The virus’s persistence in tissues post-recovery indicates potential reservoirs in extrapulmonary organs, as seen in adults and children (209, 210). This persistence has been observed in extrapulmonary organs, even in immunocompetent individuals and post-mortem subjects, several weeks or months post-infection (211). In autopsies of deceased individuals, the viral RNA was detected in various tissues, including the CNS and endothelium, weeks or months after infection despite being undetectable in plasma (212, 213). Even patients diagnosed with long COVID months or years after the initial infection exhibited lingering SARS-CoV-2 RNA in diverse tissues like the breast, appendix, and skin (213). Furthermore, in children, the virus or its remnants have been detected for weeks to months after acute infection in tissues or biological fluids, including postmortem detection of virus RNA (214). Evidence points to the presence of SARS-CoV-2 RNA in tissues of pediatric patients diagnosed with multisystem inflammatory syndrome and the detection of viral proteins and fragments in the intestine, plasma, palatine tonsils, and adenoids (215–217). It is important to note that viral RNA contaminants may be present in the analyzed samples, and their presence does not necessarily indicate persistence.

The diverse cells expressing the ACE-2 receptor can render them vulnerable to SARS-CoV-2 infection. This broad viral target might explain acute infection symptoms, yet the exact mechanism leading to prolonged symptoms remains unclear (218, 219). Although different cell targets of infection could elucidate acute symptoms, the mechanism behind the transition from acute to prolonged symptoms remains unclear (218). Extracellular vesicles (EVs) are suspected viral reservoirs, allowing SARS-CoV-2 to travel through the circulatory system, affecting endothelial cells and platelets, potentially triggering repeated immune responses and persistent symptoms (220–222).

Viral persistence, even without replication, can damage endothelial cells by inducing cytokine secretion and activating unwanted immune responses. Besides, SARS-CoV-2 can disrupt vascular homeostasis by directly infecting endothelial cells (212). Additionally, direct endothelial cell infection disrupts vascular homeostasis, impacting platelet function and promoting blood clotting and microclot attachment to the endothelium (223, 224). Furthermore, endothelial cell infection triggers increased expression of cell adhesion molecules, such as intercellular adhesion molecule 1 (ICAM-1), vascular cell adhesion protein 1 (VCAM-1), selectins (E-selectin and P-selectin), inflammatory mediators, and procoagulant factors. Endothelial dysfunction and platelet hyperactivation can lead to exposure to phosphatidylserine (PS) in the outer cell membrane. PS exposure may directly promote some procoagulant factors within the coagulation cascade and the formation of fibrinoid microclots resistant to fibrinolysis (224, 225). Meanwhile, endothelial dysfunction may increase cell permeability and leukocyte adhesion, favoring thrombus formation (212, 226).

On the other hand, it has been documented that in SARS-CoV-2 infection, mechanical damage to the microvascular glycocalyx is induced by the direct impact of fibrinogen (207). Although most recovered patients do not have detectable viral loads, the endothelial damage might stem from residual effects, possibly due to endothelial cells’ overactivation triggered by cytokine’s sustained release (68). Elevated levels of IL-1, IL-6, IFN-β, myeloid cells, and activated T cells in plasma and tissues from discharged patients are strongly associated with long COVID (227). This condition often persists with impaired oxygen exchange and tissue hypoxia, potentially contributing to a prothrombotic state and multi-organ failure in long COVID subjects (228). Moreover, hypoxia is likely to contribute to the notable and enduring B-cell abnormalities seen in cases of acute COVID-19 pneumonia (229).

### 3.6 Implications of intestinal barrier alterations in long COVID

Long COVID, recognized as a multi-system ailment (22, 108, 109), extends its impact beyond the respiratory system, affecting organs not traditionally associated with respiratory viruses. One of them is the gastrointestinal complications, which include diarrhea, vomiting, and abdominal pain (29, 230–234). Gastrointestinal involvement by substantial clinical cohorts has been reported to range from about 4% to nearly 13% in all acute COVID-19 cases (235). Significantly, a thorough investigation reported a 3.8% occurrence of viral RNA in fecal samples from patients seven months after their initial COVID-19 diagnosis (236). This trend is similarly observed in the persistent gastrointestinal complications within the spectrum of long COVID symptoms (12, 13, 22, 108).

Evidence suggests that ACE2, present in gut epithelial cells, serves as the entry point for the virus (235). The SARS-CoV-2 spike protein targets this enzyme, a crucial receptor facilitating viral entry (228, 237–240). This provides a plausible pathway for viral-induced damage. Viral RNA has been detected in stool samples, and the virus has been found to show tropism for intestinal epithelial cells, as seen in SARS-CoV-1 and Middle East respiratory syndrome coronavirus (MERS-CoV) infections (241–243). The structural similarity between the spike proteins of SARS-CoV-1 and SARS-CoV-2 and the presence of ACE2 in intestinal epithelial cells support the hypothesis of direct viral infection followed by an immune-driven inflammatory response, contributing to gastrointestinal manifestations. Studies have shown the presence of the SARS-CoV-2 nucleoprotein within the epithelial cells of hospitalized COVID-19 patients (235, 244). Another study demonstrates the presence of viruses in upper and lower gastrointestinal biopsies, which implies that gastrointestinal epithelium cells may be reservoirs of the virus even after the acute phase (245). Additionally, viruses have been identified in upper and lower gastrointestinal biopsies, indicating that gastrointestinal epithelial cells may serve as reservoirs for the virus even after the acute phase, with minimal inflammatory infiltrate observed (236, 245, 246). However, the virus in the stool does not appear to be linked to long-term COVID or the virus persistence in intestinal cells (247).

Bidirectional communication exists between the intestine and the lung (248). An intact intestinal barrier modulates pulmonary immune responses and the lung microbiome (**Figure 1**) (249, 250). In cases of acute SARS-CoV-2 infection, as in other respiratory infections, intestinal changes may be triggered due to the virus’s impact on intestinal permeability and promoting bacterial translocation (227, 248). Microbial translocation, defined as the migration of bacteria or their byproducts from the gut to extraintestinal and systemic circulation due to changes in gut mucosal integrity, can occur with or without viral infection (251). This phenomenon may impact systemic inflammation and, indirectly, affect the intestine by altering products, metabolites, and lipids associated with the intestinal microbiota (252). Numerous components of the immune system, including antiviral peptides, inflammatory mediators, immune cell chemotaxis, and secretory immunoglobulins, could be adversely affected (253). Some members of the gut microbiome produce enzymes that degrade glycans, and when these enzymes enter the circulation, they can modify the glycosylation of plasma glycoproteins, including antibodies. Glycosylation reactions regulate immune responses, including complement activation (253). Systemic inflammation, stemming from SARS-CoV-2 infection or direct viral effects on intestinal cells, can lead to changes in intestinal structure and the rupture of the epithelial barrier (250). Severely ill COVID-19 patients often display increased plasma levels of indicators of intestinal permeability, such as zonulin, a mediator of tight junctions in the digestive tract, fatty acid binding protein 2 (FABP2), lipopolysaccharide (LPS), and peptidoglycan (PGN), indicating a compromised intestinal barrier (251, 253).

Furthermore, patients with COVID-19 have consistently shown elevated levels of gut permeability and intestinal damage compared to healthy controls throughout the disease (251). The acute exacerbated immune responses triggered by SARS-CoV-2 is linked to a Th_17_ immune response, which leads to vascular permeability and leakage, resembling the immune response observed in cases of bacterial translocation (254). The available evidence suggests that the cytokine storm induced by SARS-CoV-2 is responsible for intestinal damage and may be connected to the manifestations seen in long COVID.

Intestinal damage may have connections to the manifestations observed in long COVID. Emerging evidence also hints at a potential link between gut microbiota dysbiosis and the prolonged complications of COVID‐19 (see **Figure 1**). Maintaining intestinal microbiota balance is crucial for various functions, such as extracting substrates from the diet for energy production, obtaining vitamins and short-chain fatty acids, and regulating the balance of Th_17_ and T reg responses to prevent intestinal inflammation (255, 256). Dysbiosis refers to significant alterations in microbial diversity and relative abundance (256). In individuals with severe SARS-CoV-2 infection, a relationship between intestinal microflora and lung microorganisms has been established. Opportunistic fungal and bacterial pathogens, including *Aspergillus, Candida, Actinomyces, Streptococcus, Veillonella, Rothia*, and *Clostridium*, have been found to replace commensal microbes like *Bifidobacterium romboutsia, Proteobacteria, Collinsella, Actinobacteria* and *Bacteroides* (257–259). Another study showed that changes in microbial diversity induced by inflammation during viral infection can persist for months, leading to alterations in the metabolomic profile (260). These changes involve the suppression of anti-inflammatory bacteria like *Faecalibacterium prausnitzii, Eubacterium rectale*, and *Bifidobacterium adolescentis*, and the enrichment of pathogens, including *Rothia, Erysipelatoclostridium, Ruminococcus gnavus, Ruminococcus torque*s, and *Bacteroides dorei* (260).

The persistence of microbiota dysbiosis in the gastrointestinal tract after the resolution of the disease could contribute to the prolonged symptoms, emphasizing the need to comprehend how gut microorganisms are involved in inflammation and COVID-19. Furthermore, exploring the involvement of dysbiosis in neurological disorders is essential, given the previously reported relationship between the human intestinal microbiome and the CNS (261). Alternatively, SARS-CoV-2 might infect and replicate within intestinal bacteria (262). This could lead to unique viral persistence and contribute to intestinal dysbiosis in long COVID. The bacteriological impact of SARS-CoV-2 might directly encourage the proliferation of specific bacteria, upsetting the balance in the intestinal microbiota (262).

### 3.7 Impact on the renal system

In terms of renal complications, many COVID-19 survivors, particularly those with severe pneumonia, have experienced acute kidney injury marked by a sudden decline in kidney function (263–265). About 30% of patients following acute SARS-CoV-2 infection experience renal dysfunction (266). This includes diverse conditions such as acute tubular necrosis, glomerular lesions, focal and segmental glomerulosclerosis, and renal infarction, some of which can lead to fatal outcomes (267–269). A retrospective study in China even linked kidney issues to fatal COVID-19 outcomes (244). Consequently, kidney problems have become a significant aspect of long COVID, characterized by recurrent episodes of acute kidney injury and an increased risk of chronic kidney disease (82, 91, 108, 264), as illustrated in **Figure 2**.

While there is evidence of ACE2 expression in kidney tissue, which hints at the possibility of direct viral infection (270–272), there is no confirmed kidney tropism by SARS-CoV-2, and this remains a hypothetical scenario. Other mechanisms, such as respiratory complications leading to hypoxemia and immune-driven inflammation, may contribute to kidney manifestations (273, 274). It is important to note that just as kidney injury raises the risk of fatal COVID-19 (38, 44, 275), the reverse relationship is observed: COVID-19 heightens the risk of developing chronic kidney disease (91). These findings underscore the importance of preparing healthcare systems for potential increases in kidney disease cases as long as COVID affects individuals in the months ahead.

## 4 Reactivation of latent infections

Reactivation of latent viral infections, including Epstein-Barr virus (EBV), human herpesvirus 6 (HHV-6), and human cytomegalovirus (HCMV), is a common occurrence in states of compromised immunity. Human herpesviruses like EBV and HHV-6 are prevalent globally, with over 90% of adults carrying one or more of these viruses (276, 277). Among the risk factors for viral reactivation are stress and immunosuppression, and also this reactivation has been documented in severe illness, particularly among ICU patients (278). Patients with COVID-19, whether experiencing mild or severe cases, have reported the reactivation of latent herpesviruses (279–282). However, the reactivation tends to be more severe in individuals with a critical illness and less pronounced in those with milder cases (279–282). The stress and the notable increase in cytokines and other inflammatory molecules is mainly caused by viral proteins, including ORF8, a unique accessory protein specific of SARS-CoV-2 are believed to contribute to the reactivation of these viruses (283, 284). Furthermore, this reactivation may enhance SARS-CoV-2’s entry into cells, potentially increasing viral load and symptom severity (285). While causal links have not been definitively established, it has been speculated that persistent EBV, HHV-6, and SARS-CoV-2 infections could fuel the chronic inflammation associated with long COVID (281, 286, 287). A study that compared patients with long COVID with 1 or more than 5 symptoms to those without long COVID found higher Epstein-Barr virus nuclear antigen (EBNA) IgG levels. More specifically, these patients have more neurocognitive symptoms (276). Interestingly, long COVID patients who reported preexisting autoimmune diseases like thyroiditis and have experienced fatigue showed higher levels of early antigen diffuse component (EA-D) IgG (276).

Additionally, SARS-CoV-2 has been suggested as a risk factor for various cancers (288), raising the possibility that latent herpesvirus reactivation may play a role in these oncogenic processes. This emphasizes the need to closely monitor reactivated viral infections in patients with current or past SARS-CoV-2 infections.

Moreover, the reactivation of infections has been associated with neurological complications. Long COVID shares similarities with myalgic encephalomyelitis/chronic fatigue syndrome (ME/CFS) due to comparable manifestations (289). ME/CFS is characterized by systemic exertion intolerance with neurological and immunological components, and viral infections can be a trigger (289, 290). In survivors of SARS-CoV-1, 27% reported ME/CFS symptoms four years after infection, a trend observed in long COVID patients (290). Opportunistic viruses like EBV, HHV-6, and HCMV have been linked to the development of ME/CFS (291). Consequently, reactivation of these viruses has been associated with long COVID symptoms (292, 293). A study found that long COVID patients had higher EBV nuclear antigen (NA) immunoglobulin G (IgG) levels compared to those without the condition (293). Also, EBV early antigen-diffuse (EA-D) IgG was correlated to long COVID manifestations, with fatigue and insomnia being the most frequently reported (292). Furthermore, the reactivation of EBV and HHV-6 was detected in long COVID patients who reported symptoms like sleep disorders, persistent cough, anosmia/ageusia, hair loss, shortness of breath, and chest pain (294).

Reactivation of latent infections related to COVID-19 extends beyond viral diseases. Notably, cases of tuberculosis (TB) reactivation following SARS-CoV-2 infection have been documented (295–299). The possible immunosuppressive effects of the disease, which remains controversial, and the treatments may contribute to infectious disease reactivation. Alarmingly, the WHO’s annual tuberculosis report indicates an increase in TB incidence since the beginning of the pandemic, exacerbating a condition that already imposes a significant global healthcare and economic burden (300).

## 5 Immune response during long COVID

Understanding the immune response to long COVID necessitates insight into the acute phase of the disease. The host immune reaction, encompassing innate and adaptive components, plays a crucial role in COVID-19. This involves the release of substantial quantities of pro-inflammatory cytokines and inflammatory cells such as neutrophils and macrophages (155, 301, 302) along with the generation of virus-specific CD4^+^ T cells, CD8^+^ T cells, and B cells (303–308). The impact of this immune response on the clinical manifestations of COVID-19 has been reported. Clinical research has indicated that in numerous severe cases, immunopathology —organ and tissue damage resulting from an imbalanced immune response—may be a primary factor in life-threatening injuries for patients (150, 301, 309–314). Acute exacerbated immune responses, mainly the self-sustaining inflammatory chain reaction are commonly observed in critical COVID-19 cases (301, 303, 309, 310). Additionally, markers of inflammation in the blood linked to immunopathology and multi-organ damage have been identified, including interferons, neutrophil markers, and other inflammatory cytokines (314). Immunopathology has also been demonstrated to play a substantial role in severe acute pediatric cases, with a relatively common manifestation being the severe, and at times fatal, systemic inflammatory condition known as multi-system inflammation in children (MIS-C). This syndrome involves exacerbated respiratory and cardiovascular tissue inflammation accompanied by fever (264, 265, 270–274).

Persistent immune dysregulation is suspected to play a role in specific chronic manifestations of long COVID (**Figure 2**) (24). Evidence indicates that patients exhibiting these manifestations display altered immune mediators, both cellular and humoral, with a notable focus on modified T-cell populations (21, 104, 108). It is plausible to hypothesize that these altered populations may contribute to persistent immunopathology, potentially accounting for long-term symptoms characterized by inflammation, such as chronic fever and muscle and joint pain (20, 164, 311, 312). However, it is crucial to emphasize that the connection between acute and chronic immune regulation post-SARS-CoV-2 infection is not yet fully elucidated.

Clinical studies utilizing blood samples from COVID-19-convalescent volunteers have demonstrated that SARS-CoV-2 infection strongly induces coronavirus-specific T lymphocytes and memory B cells (315–319). Besides, evidence suggests that these lymphocytes may remain persistently elevated, potentially explaining at least some clinical manifestations of long COVID (113, 150). Additionally, it has been observed that in COVID-19-convalescent patients, there are broad alterations in the transcriptome of peripheral blood mononuclear cells (PBMCs) in those with clinical presentation compatible with long COVID (320). Significant differences in innate and adaptive immune cell populations in COVID-19 convalescent individuals compared to uninfected controls were observed (320). Furthermore, a clinical study employing a high-dimensional characterization of the lungs of COVID-19 convalescent subjects found an enrichment of T and B cells in the respiratory tract compared to blood levels (110). Additionally, it was observed that the gastrointestinal manifestations of long COVID were linked to the expansion of SARS-CoV-2-specific CD8^+^ and CD4^+^ T cell populations (41).

Dysregulated tissue-resident T cells have been suggested as potential drivers of chronic lung pathology post-respiratory infections (321, 322), implying that long COVID pathology may stem from a dysregulated cellular response (315). CD4^+^ T cell responses during COVID-19 are correlated with disease severity, suggesting that a more severe acute SARS-CoV-2 infection could lead to a greater degree of T cell response dysregulation, potentially explaining chronic manifestations (24). Conversely, there was no correlation between immune dysregulation and persistent viral load (24), indicating that COVID-19 induces alterations in cell immunity regardless of chronic viral load, which could be a key pathophysiological trigger of long COVID (320, 323).

On the other hand, studies have shown that COVID-19 can induce significant decreases in several components of the cellular immune response, including cytokines, complements, and immune cells (303, 324, 325). Recent research showed marked reductions in counts and percentages of total lymphocytes, total T cells, CD4^+^ T cells, CD8^+^ T cells, B cells, and natural killer cells (NK) without changes in the typical TCD4:TCD8 ratio of approximately 1:1 in healthy subjects whether vaccinated or unvaccinated, who are COVID-19 survivors (324) (**Figure 1**). Substantial dysregulations in CD8^+^ T cells expressing degranulation markers have been reported after SARS-CoV-2 infection (24). Furthermore, a machine-learning-assisted clinical characterization of the immune response of healthy volunteers, COVID-19 patients, and long COVID patients revealed significantly elevated B cell and CD14^+^ CD16^+^ CCR5^+^ monocyte levels, along with reduced T regulatory cells (Treg) and programmed cell death protein 1(PD-1)-expressing CD4^+^ and CD8^+^ T cells in the long COVID group compared to healthy controls (325). Moreover, immunological characterization of long COVID patients found a persistent increase of CD16^+^ monocyte levels and sustained expression of the SARS-CoV-2 S1 protein in CD14lo, CD16^+^ monocytes (326). While the precise role of this expression remains uncertain, it is speculated that it could contribute to chronic vascular inflammation (326). These observations may suggest that if these alterations in cellular immunity persist, they could at least partially contribute to the symptoms present in long COVID. Consistent with this, significantly higher SARS-CoV-2-specific IFN-γ-producing CD4^+^ and CD8^+^ T cells were found in subjects with prior pulmonary disease, indicating that this condition may lead to a higher likelihood of future persistent cellular immune dysregulation after experiencing COVID-19 (24). Furthermore, a study found increased levels of activated CD4^+^ and CD8^+^ T-cells after 3 months of recovery from mild, moderate, and severe COVID-19. Higher plasma levels of T-cell-related IL-4, IL-7, IL-17, and tumor necrosis factor-alpha (TNF-α) were observed to compared mild and moderate patients (327). Despite all this evidence, a study evaluating SARS-CoV-2 cellular immunity in health workers with confirmed infection, with or without persistent symptoms, found no differences in viral neutralization or T-cell responses (328). Recently, it was observed that T cells from COVID-19 patients exhibited significantly elevated levels of IL-2 production in response to stimulation with SARS-CoV-2 peptides compared to an unexposed control group (329).

SARS-CoV-2 infection triggers a strong induction of virus-specific B cells, resulting in abundant production of IgG antibodies primarily targeting the receptor-binding domain of the spike protein (S-RBD) and the nucleocapsid protein (NP) (308, 317, 330). Studies of individuals recovering from COVID-19 consistently show elevated IgG levels persisting for up to 24 weeks post-infection (315, 320). These humoral responses correlated with disease severity and the number of post-acute symptoms (20, 320, 331). Similar patterns emerge in cellular immunity. However, the precise implications of the upregulation of both humoral and cellular responses in the pathogenesis of long COVID remain unclear. Notably, some studies find no clear correlation between the humoral response and the incidence and severity of long COVID manifestations (108, 328). It is crucial to note that elevated antibody titers do not always indicate the presence of symptoms. COVID-19 vaccinations have been associated with a sustained upregulation of both humoral and cellular immune responses, with a notable absence of chronic adverse effects in most cases (54, 78, 80, 96, 332–338). Interestingly, comorbidities, particularly diabetes, chronic heart disease, and hypertension, correlate with higher convalescent antibody titers after COVID-19 (20).

On the other hand, alterations have been observed in the development of B cells, wherein antibody-secreting cells are produced extrafollicularly (339, 340). This pathway has been previously described in individuals with lupus (341). While responses directed against the virus are initiated, they can also give rise to autoreactive humoral responses (autoantibodies), which persist for months in individuals experiencing post-COVID-19 sequelae (339, 340). Autoantibodies have emerged as potentially significant contributors to COVID-19 severity (41). Reports suggest these molecules might be responsible for up to a fifth of COVID-19-related deaths (342). Autoantibody concentrations have been positively correlated with age and the male sex, potentially explaining the higher severity and case-fatality ratio observed in these (342, 343). Additionally, 10% of individuals with acute COVID-19 infections had significant concentrations of autoantibodies that directly inhibit type-I IFN (342), but these antibodies were not found in significant concentrations in asymptomatic COVID-19 patients (343).

Like in the acute phase of COVID-19, autoantibodies in long COVID patients have been reported (344). A comprehensive multi-omics study reported the presence of autoantibodies predate SARS-CoV-2 infection and can be one of many factors predicting the incidence of long COVID (**Figure 1**) (40). Recent multiparameter analyses of long COVID highlight the significance of specific autoantibody production as a predictor among various risk factors for long COVID incidence (40). Furthermore, persistent circulation of the SARS-CoV-2 spike protein has been reported, potentially serving as a mechanistic link to long COVID symptoms (65, 67). Another category of autoantibodies targets G-protein coupled receptors (GPCRs), which have been implicated in cardiovascular and neurological development (345). In long COVID patients, α1 adrenergic receptor (α_1_ AdR), β_1_ AdR, and β_2_ AdR and the muscarinic acetylcholine receptors M_2_ (M_2_ AChR), M_3_ AChR, and M_4_ AChR were the most found and correlates with the severity of the neurological symptoms (345–347).

Despite these findings, available data on the humoral response during long COVID fail to provide conclusive evidence that can significantly contribute to our understanding of the pathophysiological mechanisms underlying this condition, underscoring the need for further research.

## 6 Discussion

Long COVID is a newly recognized clinical condition, and numerous uncertainties persist. Consequently, investigations into this emerging condition are only beginning to uncover potential underlying mechanisms (23, 24, 65, 67, 110, 112, 266, 302, 308, 315, 316, 323, 348). Public health authorities like the CDC actively conduct short-term and multi-year studies to enhance our understanding of this condition. Available data underscores that this clinical phenomenon is intricate and diverse, affecting multiple bodily systems and exhibiting persistence over months to years in some instances, summarized in **Table 1** (23, 49, 139, 151, 164, 320, 350). As mentioned earlier, the definition of persistent symptoms for the post-COVID diagnosis is crucial in discerning whether they are indicative of long COVID or if the recovery process is simply slower than anticipated. Prolonged inflammation has been proposed as a potential driver of long-term manifestations following COVID-19. Indeed, the role of sustained inflammation in developing cardiovascular, respiratory, immunological, and neurological complications after COVID-19 has been elucidated (23, 91, 110, 112, 155, 355). National healthcare systems should be understanding the likelihood that some COVID-19 survivors may transition into patients with chronic disabilities. Simultaneously, the possibility of chronically ill COVID-19 survivors potentially contributing to the emergence of novel variants of concern due to persistent viral loads necessitates careful attention (66, 71). Current evidence on the existence of different subtypes of long COVID remains inconclusive. However, two studies have progressed in subtyping this condition (339, 358). Using semantic phenotypic grouping, the first study successfully stratified the disease into six distinct groups (358). These groups exhibit unique profiles of phenotypic abnormalities, encompassing specific pulmonary, neuropsychiatric, and cardiovascular manifestations, with one group exhibiting extensive and severe symptoms correlated with increased mortality (358). The second study, employing proteomic techniques, further subclassifies long COVID into two conditions. These conditions are characterized by the presence or absence of broad inflammatory signatures indicating increased neutrophil activity and qualitative changes in memory and B cell responses (339). Characterizing and subclassifying long COVID can potentially improve the diagnosis and treatment strategies for this ailment. Therefore, long-term studies must delve into the epidemiology, mechanisms, pathophysiology, and possible management of long COVID to address this emerging crisis with the utmost diligence from healthcare institutions. Concurrently, public health organizations should formulate comprehensive management strategies that recognize the diverse manifestations characteristic of it and address its manifold consequences.

**Table 1:** Epidemiological, clinical, pathophysiological, and immunological characteristics of long COVID.

Feature	Available facts	References
**Diagnosis**	The US CDC defines long COVID as any lingering COVID-19 symptom four weeks after initial infection.	(12)
**Incidence**	Estimates vary between 50 and 70% of COVID-19 hospitalized cases, with 20% being an increasingly accepted estimate (as per the study by Bull-Otterson et al.). Prevalence among non-hospitalized patients has been reported to be 10-30% and only 10-12% of the vaccinated cases. The incidence depends on whether the study considers symptoms after 4 weeks after the initial diagnosis.	(17–24, 27)
**Effects of vaccination**	Vaccination provides only modest protection against long COVID incidence in case of breakthrough SARS-CoV-2 infection. Limited case report data suggest vaccination may improve symptoms of long COVID patients and help clear chronic infections, but some reports are conflicting.	(74, 82–84, 90, 92, 101, 104)
**Duration**	Variable. Known case durations range from weeks to months and possibly years, with some cases that emerged early during the pandemic still experiencing lingering symptoms at this publication.	(17, 28, 32)
**Risk factors**	Diabetes, cardiovascular disease, inflammatory conditions, old age, and severe COVID-19 have evidence of association, but conflicting data exist.	(19, 21, 33, 37, 38, 40–42, 46–49)
**Viral involvement in the condition**	The condition may occur regardless of chronic infection with SARS-CoV-2, which is defined as persistently detectable viral genetic material. The latter is suspected of facilitating the emergence of novel variants of concern.	(12, 23, 63, 64, 66, 69–73)
**Cardiovascular complications**	Myocardial inflammation and magnetic resonance abnormalities are common among COVID-19 survivors with lingering symptoms. SARS-CoV-2 invasion of cardiac tissue has been described.	(149, 198, 200–203, 349)
**Respiratory manifestations**	Dyspnea and shortness of breath are the most common symptoms in long COVID patients. Respiratory involvement seems to correlate with neurological abnormalities. The incidence of pulmonary fibrosis has been described in several cases, and it correlates with local upregulation of inflammatory markers and inflammasome activation.	(91, 137–139, 141, 143, 144, 156, 314, 350)
**Neurological alterations**	A wide array of neurological complications is often called “neuro-COVID.” It is characterized chiefly by brain fog. SARS-CoV-2 interaction with the BBB and invasion of the CNS have been described. The emergence of GBS syndrome has been associated with cases of SARS-CoV-2 infection.	(5, 141, 165, 175, 176, 178, 179, 181, 182, 184, 189–192, 324, 331, 351)
**Damage to endothelial barriers**	Endothelial damage can occur due to viral persistence or due to the ability of SARS-CoV-2 to infect endothelial cells. The disturbance of homeostasis can affect platelet function, blood coagulation, cell permeability and leukocyte adhesion, favoring the formation of thrombi. Which may contribute to multiple organ failure in subjects with long COVID.	(212, 223, 224, 226, 228)
**Intestinal barrier alterations**	ACE2 present in gut epithelial cells, could serve as the entry point for the virus. SARS-CoV-2 might infect and replicate within intestinal bacteria. The virus’s could impact on intestinal permeability and promoting bacterial translocation. Changes in microbial diversity induced by inflammation can lead to alterations in the metabolic profile. Changes in the microbiota could precede the development of long COVID and exacerbate inflammatory processes and precipitate multiorgan alterations.	(45, 233, 235, 248, 264, 265)
**Alterations in other body systems**	Renal and hepatic manifestations have been reported as significant sequelae of COVID-19. Mechanisms for these complications are unclear, but viral tropism has emerged as a hypothesis due to the presence of ACE2 in these tissues. Respiratory complications, hypoxemia and immune-driven inflammation may contribute to kidney manifestations.	(264, 273, 352)
**Reactivations of latent infections**	EBV, HHV-6, and HCMV reactivation have been described in long COVID patients and are associated with neurological complications such as ME/CFS. TB reactivation has been described in patients who recovered from COVID-19.	(280, 282, 292, 294, 297, 299, 353)
**Changes in immunity**	Natural infection may mediate some chronic symptoms by strong induction of virus-specific CD4^+^ and CD8^+^ T and B cells. Significant alterations in T, B, and NK cell quantities have been reported in COVID-19 survivors. Consistently elevated IgGs have been described in long-COVID patients. Autoantibodies may mediate both severe disease and chronic inflammation in some long COVID cases. Persistent circulation of the SARS-CoV-2 spike protein could be a putative antigen mediating chronic manifestations.	(23, 67, 110, 150, 307, 315, 316, 318, 320, 342, 343, 354)
**Changes in inflammation**	A state of hyperinflammation has been linked to the long COVID condition. This condition may be partly due to mast cell activation. Hyperglycemia and new-onset diabetes have been proposed as possible consequences of excessive inflammation during a SARS-CoV-2 infection.	(23, 38, 112, 135, 140, 151–153, 156, 203–205, 274, 302, 348, 354–357)

## Author contributions

KB: Conceptualization, Writing – original draft, Writing – review & editing. BD-V: Conceptualization, Writing – original draft, Writing – review & editing. LR-G: Writing – review & editing. TR: Writing – review & editing. CR: Writing – review & editing. PG: Writing – review & editing. AK: Conceptualization, Writing – review & editing.
